# Neurogenic Voiding Dysfunction in Spinal Cord Injury and Stroke: Urodynamic Evaluation, Functional Classification, and Therapeutic Strategies

**DOI:** 10.7759/cureus.89348

**Published:** 2025-08-04

**Authors:** Enmanuel Sevilla Torres, Edgardo J Soto-Junco, Santiago Daniel Baizan Orias, Alberto Rojas Peláez, David Sáenz Araya, Freddy Lizano Guevara

**Affiliations:** 1 General Medicine, Universidad de Ciencias Médicas (UCIMED), San José, CRI; 2 General Medicine, Universidad de Costa Rica, San José, CRI

**Keywords:** neurogenic urinary bladder, spinal cord injuries, stroke, urinary incontinence, urodynamics

## Abstract

Many patients with injuries to their central nervous system, especially those who have had a stroke or a spinal cord injury, have neurogenic voiding dysfunction. It happens when patients can't control their bladder voluntarily, which can make them feel urgency, experience leaks, or have trouble emptying their bladder. If not treated right away, it can cause infections, damage to the bladder, or even kidney problems over time.

This review will explain how this type of dysfunction works, how it is diagnosed, and what treatment options are available. It will mostly focus on stroke and spinal cord injuries. To do this, recent studies that were published between 2019 and 2025 on PubMed, Scopus, and ScienceDirect were reviewed. The main focus was on studies that were clinically relevant and had strong methods.

Symptoms may vary based on which part of the nervous system is affected. Injuries that are higher up the spinal cord tend to cause an overactive bladder and poor coordination between the sphincter and bladder, while injuries on a lower spinal cord level, particularly sacral ones, often lead to an unreactive bladder. A diagnosis is generally made through a physical exam and diagnostic tests, including urodynamics, uroflowmetry, and ultrasound.

For treatment, options include clean intermittent catheterization, antimuscarinic or botulinum toxin medications, neuromodulation, and, in severe cases, surgery. Regular follow-ups are crucial to avoid complications such as kidney failure, kidney stones, or infections.

Managing this condition requires a collaborative approach. Nurses, therapists, neurologists, urologists, and rehabilitation specialists all play a crucial role in aiding patients to improve their quality of life and prevent further health complications.

## Introduction and background

Neurogenic lower urinary tract dysfunction (NLUTD) arises from damage to the neural pathways responsible for controlling micturition, leading to symptoms such as urinary incontinence or urgency [1]. This condition commonly occurs in the context of neurological diseases such as stroke (CVA) and multiple sclerosis, among others, due to compromised voluntary bladder control [2,3].

NLUTD is a frequent condition in patients with spinal cord injury (SCI), placing them at increased risk for urinary tract infections and renal impairment, which can negatively impact their quality of life [4,5]. Neurostimulation and pharmacological treatments have been proposed to reduce complications and improve bladder function [6,7].

From an epidemiological perspective, a large proportion of individuals with SCI develop chronic urinary dysfunction over time, which may vary depending on the type and severity of the injury [4]. A 2024 study showed that personalized treatment approaches are needed depending on the patient’s clinical and social context [8].

NLUTD is also intrinsically associated with stroke, particularly in the form of urinary incontinence, which increases the risk of infection and mortality [9]. Overactive bladder and incontinence are common in these individuals, and treatment often involves solifenacin and trospium, which have shown the best results to date; however, further studies are needed to establish the optimal therapeutic strategy [7].

The objective of this review is to describe the pathophysiological mechanisms, the evolution of diagnostic approaches, and the main treatments for managing urinary dysfunction in patients with neurological injuries, especially those with SCI or stroke.

Methodology

This review was based on a meticulous search aimed at generating an up-to-date summary of urinary disorders associated with neurological injuries, with a particular emphasis on SCI and cerebrovascular accident (CVA). To achieve this objective, three widely recognized biomedical databases were examined: PubMed, Scopus, and ScienceDirect, focusing on publications released between 2019 and 2025, including texts in both English and Spanish. The combination of search terms included precise terminology such as neurogenic bladder, lower urinary tract dysfunction, spinal cord injury, stroke, urinary incontinence, and urodynamic assessment. These terms were carefully refined using Boolean operators to improve the specificity of results relevant to clinical, diagnostic, and therapeutic topics.

Included materials consisted of original research articles, systematic reviews, and consensus documents from accredited scientific organizations. Publications or redundant studies that did not provide relevant data for practical or broadly applicable clinical contexts were excluded.

It is important to acknowledge that the drafting of this manuscript involved the use of artificial intelligence as an auxiliary tool, which contributed to improving the structure, synthesis, and coherence of the text without replacing the clinical insight or academic analysis provided by the authors.

## Review

Normal physiology of bladder control

To control urinary micturition, coordinated interaction between the central and peripheral nervous systems is required. At the cortical level, the urge to urinate is suppressed until a socially appropriate moment, a function mediated by the prefrontal cortex. When this region is impaired, it can result in urge incontinence [10]. In the brainstem, the micturition reflex is initiated via the pontine micturition center, which receives input from the periaqueductal gray matter, thereby coordinating urethral sphincter relaxation and detrusor muscle activation [11].

The spinal cord acts as a bridge between the bladder and the brain, integrating sensory stimuli and generating motor reflex responses that regulate bladder storage or emptying [12]. Parasympathetic nerves induce detrusor contraction at the peripheral level, whereas sympathetic nerves promote urine storage by relaxing the bladder and contracting the urethral outlet [13].

Effective micturition requires precise coordination between the detrusor muscle and the sphincter: as one contracts, the other must relax. This synergy is regulated by both the central and peripheral nervous systems [12]. Additionally, brain networks such as the central executive, salience, and attentional networks integrate sensory input and modulate the micturition response based on the individual's context [14].

Through postnatal neurological development, individuals acquire the ability to control the bladder as the nervous system progressively matures. Therefore, following a neurological injury, neuroplasticity plays a key role in the rehabilitation of bladder control in patients with spinal cord injury or stroke by enabling functional reorganization [15].

Pathophysiology of neurogenic voiding dysfunction

SCI occurring above the S2-S4 segments can result in neurogenic detrusor overactivity, leading to incontinence, urgency, and even involuntary contractions. This is due to the loss of supraspinal inhibitory control [1,16]. This condition is often accompanied by detrusor-sphincter dyssynergia (DSD), characterized by increased intravesical pressure and incomplete voiding caused by a lack of coordination between the bladder and the sphincter [17,18].

In cases where the lower spinal cord or cauda equina is affected, a hypocontractile or underactive bladder may develop, leading to urinary retention due to weak or absent detrusor contractions as a result of damage to the sacral micturition center [4,19]. DSD may also be present in these cases, further complicating bladder emptying, although it is less common in this context [19].

In patients with stroke, overactive bladder is frequently observed due to loss of cortical control caused by lesions in the dominant hemisphere. In contrast, involvement of the non-dominant hemisphere is associated with milder urinary symptoms [20].

Clinical and functional classification

Neurogenic bladder can be primarily classified as a reflex or flaccid bladder. Reflex bladder is associated with upper motor neuron lesions and manifests as incontinence and involuntary detrusor contractions. In contrast, flaccid bladder occurs in cases of lower motor neuron lesions and is characterized by absent bladder contractions, fluid retention, and incomplete bladder emptying [19].

These conditions can pose significant clinical risks, as urinary incontinence is common and may negatively impact quality of life. Only 50% of affected children achieve continence, with surgical approaches being the most effective in such cases [21]. On the other hand, intermittent catheterization can be used to manage incomplete bladder emptying in patients with a flaccid bladder [8].

Urinary tract infections (UTIs) are common due to catheter use and residual urine; prevention requires prudent antibiotic management and the implementation of new strategies [22]. Vesicoureteral reflux is also a serious complication, detectable by video urodynamic studies and strongly associated with renal damage [23].

Likewise, the use of catheters can lead to UTIs due to urine retention. Preventing this requires careful antibiotic use and innovative strategies [22]. Vesicoureteral reflux may result in significant complications, but it can be detected through video urodynamic testing and is primarily linked to kidney damage [23].

Treatment options include intermittent catheterization, although its use is limited (only 19.3% of patients use it regularly) [8]. Antimuscarinic agents and botulinum toxin are also used to reduce symptoms [24]. Surgical interventions such as bladder augmentation are reserved for refractory cases due to their considerable risk of complications [21].

Diagnostic evaluation

The diagnostic evaluation of patients with voiding dysfunction due to neurological injury begins with a detailed medical history. It is essential to distinguish between irritative symptoms, such as urinary frequency or urgency, and obstructive symptoms, which involve decreased urinary flow or hesitancy. This differentiation allows clinicians to define the type of lower urinary tract dysfunction by guiding the diagnosis toward specific patterns of neurological involvement [25,26].

A key aspect during patient history-taking is the neurological background. It is important to determine whether the patient has suffered from SCI, traumatic brain injury, neurodegenerative diseases, or any other previous condition affecting the nervous system. These conditions may cause either acute or chronic disruption of bladder control and must be considered when analyzing the current symptoms [20]. In addition, it is crucial to identify prior treatments, both surgical and pharmacological, as they provide valuable insights into the clinical course and the efficacy of past interventions, enabling the development of the most appropriate therapeutic strategy [27].

During the physical examination, attention should be focused on evaluating the sacral reflex arc, which is responsible for neuromuscular control of the pelvic floor and sphincters. Assessing the anal and bulbocavernosus reflexes helps determine the functional integrity of the sacral segments of the spinal cord. Alterations in these reflexes may indicate neurological lesions affecting the urological system. Additionally, evaluating perineal muscle tone is important to identify potential neuromuscular dysfunctions that could impair continence mechanisms [28].

Complementary paraclinical studies provide an objective assessment of lower urinary tract function. Renal and bladder ultrasound can detect structural abnormalities and measure post-void residual urine volume [25,29]. Uroflowmetry, on the other hand, records urinary flow patterns and can provide evidence of detrusor-sphincter dyssynergia or functional obstruction [29].

A cornerstone of functional diagnosis is urodynamic testing, which offers detailed information about intravesical pressure, bladder capacity, and the behavior of the detrusor muscle and urethral sphincter. This test is critical to confirm the presence of NLUTD and to guide the clinician toward the most suitable treatment plan [25,29]. Finally, in selected cases, cystoscopy may be employed, particularly when anatomical abnormalities of the bladder or urethra are suspected as contributing factors to the urinary symptoms [29].

Therapeutic management

An integrated approach is required to effectively manage neurogenic bladder dysfunction. Ensuring treatment adherence depends largely on patients and their caregivers being well educated and informed, particularly regarding catheterization and the early detection of UTI symptoms [30,31]. Likewise, regular urine analysis, ultrasound imaging, and urodynamic studies allow for continuous monitoring of renal status and early detection of bladder deterioration [31,32].

To improve urinary control and reduce urgency and incontinence, conservative treatment may include bladder retraining and pelvic floor exercises [33]. Nevertheless, clean intermittent catheterization remains a cornerstone of treatment, especially in patients with spina bifida or spinal cord injury, as it facilitates complete bladder emptying and reduces the risk of infection [30,31]. Adjusting voiding schedules and modifying fluid intake can also help prevent bladder overdistension [5].

Pharmacologic treatment includes the use of antimuscarinic agents and beta-3 agonists, such as oxybutynin and mirabegron, aimed at controlling detrusor overactivity [29,31]. In contrast, in cases of urinary retention, alpha-blockers and cholinergic agonists are used to promote bladder emptying [29]. For patients who have suffered a stroke, careful evaluation of drug interactions and tolerability is necessary to prevent neurological side effects [26].

Botulinum toxin injections have proven to be highly effective in reducing detrusor overactivity, although they may be associated with adverse effects [34]. Sacral neuromodulation offers an alternative for refractory cases, as it modulates the neural pathways that control the bladder [33]. Finally, urinary diversions may be considered to preserve renal function and improve quality of life in more complex situations [35].

Among the most common complications are urinary tract infections, which can be prevented through proper catheterization techniques, rational antibiotic management, and the use of botulinum toxin [5,30].

Complications and follow-up

One of the most common complications in patients with neurogenic bladder is urinary tract infections (UTIs), which increase with the use of catheters and impaired bladder emptying. In patients with spinal cord injury (SCI), autonomic dysreflexia, detrusor-sphincter dyssynergia, and vesicoureteral reflux are factors that significantly raise the risk of recurrent UTIs [23]. Furthermore, persistent bacterial colonization becomes more likely and frequent when the barrier function of the bladder mucosa is disrupted [36]. Although rational antibiotic use and clean intermittent catheterization are core elements of management, UTI prevention remains a clinical challenge [22].

The development of chronic kidney disease is one of the most serious complications, typically associated with reduced bladder capacity and a combination of incomplete emptying and detrusor overactivity [23]. It is essential to maintain low intravesical pressures and ensure complete bladder emptying in order to preserve renal function [37]. As previously mentioned, intravesical botulinum toxin injections and antimuscarinic medications are used to control detrusor activity and protect the upper urinary tract [24].

Structural bladder damage and urinary stone formation are also significant risks in a neurogenic bladder. Urinary stasis and incomplete emptying promote stone formation [37], while elevated pressures during voiding may lead to morphofunctional changes such as urolithiasis and bladder fibrosis [23]. In severe cases, reconstructive surgeries such as bladder augmentation may be necessary to prevent further complications [24].

Periodic renal and urodynamic monitoring is essential for early detection of complications. Tests such as cystometry and pressure-flow studies allow clinicians to adjust treatment based on the functional evolution of the lower urinary tract [29]. Likewise, renal ultrasound helps assess the condition of the upper urinary tract [24]. Given the complexity of these cases, a multidisciplinary approach involving specialists such as urologists and neurologists is strongly recommended to achieve optimal clinical outcomes [29].

Multidisciplinary approach

In order to proficiently address voiding dysfunction in individuals afflicted with neurological injuries, a synergistic, multidisciplinary framework is imperative. Experts from diverse domains must collaboratively engage and disseminate their specialized knowledge. Within this framework, the neurologist assumes a pivotal function in assessing the neurological facets of urinary disorders, especially in instances such as multiple sclerosis, wherein the central nervous system is intrinsically implicated. Their contributions are indispensable for discerning the ways in which neurological manifestations and their corresponding treatments influence urinary functionality, thereby facilitating prompt diagnosis and appropriate referrals to additional specialists when warranted [29,38].

Urologists are conventionally tasked with the oversight of neurogenic lower urinary tract dysfunction. Their responsibilities encompass executing fundamental diagnostic assessments such as urodynamic studies, which inform therapeutic decisions and assist in formulating strategies aimed at preserving bladder functionality and averting potential complications. Urologists also provide both conservative and surgical interventions aimed at preventing urinary incontinence and mitigating the risk of infections [32,38,39].

The role of the physiatrist is to facilitate the comprehensive recovery of the patient. This professional formulates individualized rehabilitation plans that encompass both physical and psychological health recovery. Physiatrists strive to ensure that safe and efficacious treatments for neurogenic bladder align with overarching rehabilitation objectives, with the aim of maximizing patient autonomy [39,40].

Nurses play a crucial role in the daily management of bladder dysfunction. They assist patients and their families in adapting to treatment regimens by providing essential information, emotional support, and adherence to care protocols. Their continuous presence ensures the monitoring of potential complications and the compliance with therapeutic interventions [29].

Occupational therapists play an important role in helping people with urinary dysfunction maintain and improve their ability to do everyday activities. When someone has trouble with bladder control, these professionals help adapt the home environment, provide functional training, and guide the selection of assistive devices that make bladder management easier at home [40].

Integrated functional rehabilitation is also a key part of treating a neurogenic bladder. These programs combine physical therapy, occupational therapy, and patient education to help restore lost function and improve quality of life. What stands out is that each plan is personalized, depending on how much neurological damage there is and what specific limitations the person has [40].

## Conclusions

Neurogenic bladder dysfunction is a fairly common and potentially serious complication in people who have suffered spinal cord injuries or strokes. The way it presents depends a lot on how severe and where the neurological damage is, which is why it’s important to identify whether the bladder is overactive, underactive, or if there’s poor coordination between the detrusor muscle and the sphincter. Getting an accurate diagnosis of lower urinary tract dysfunction in these cases requires a full clinical evaluation, supported by tools like urodynamic studies and kidney ultrasounds. It’s important that specialists like neurologists, urologists, physiatrists, nurses, and occupational therapists work together and share what they know so the patient gets the most complete and effective care possible.
